# The Prevalence of CD146 Expression in Breast Cancer Subtypes and Its Relation to Outcome

**DOI:** 10.3390/cancers10050134

**Published:** 2018-05-05

**Authors:** Ingeborg E. de Kruijff, Anna M. Timmermans, Michael A. den Bakker, Anita M.A.C. Trapman-Jansen, Renée Foekens, Marion E. Meijer-Van Gelder, Esther Oomen-de Hoop, Marcel Smid, Antoinette Hollestelle, Carolien H.M. van Deurzen, John A. Foekens, John W.M. Martens, Stefan Sleijfer

**Affiliations:** 1 Department of Medical Oncology, Erasmus MC Cancer Institute, ‘s-Gravendijkwal 230, 3015 CE Rotterdam, The Netherlands; a.timmermans@erasmusmc.nl (A.M.T.); a.trapman@erasmusmc.nl (A.M.A.C.T.-J.); r.foekens@erasmusmc.nl (R.F.); mevgelder@hotmail.com (M.E.M.-V.G.); e.oomen-dehoop@erasmusmc.nl (E.O.-d.H.); m.smid@erasmusmc.nl (M.S.); a.hollestelle@erasmusmc.nl (A.H.); j.foekens@erasmusmc.nl (J.A.F.); j.martens@erasmusmc.nl (J.W.M.M.); s.sleijfer@erasmusmc.nl (S.S.); 2 Department of Pathology, Erasmus MC Cancer Institute, ‘s-Gravendijkwal 230, 3015 CE Rotterdam, The Netherlands; m.denbakker@erasmusmc.nl (M.A.d.B.); c.h.m.vandeurzen@erasmusmc.nl (C.H.M.v.D.)

**Keywords:** breast cancer, CD146, EMT, tamoxifen resistance, prognostic marker

## Abstract

CD146, involved in epithelial-to-mesenchymal transition (EMT), might affect cancer aggressiveness. We here investigated the prevalence of CD146 expression in breast cancer subtypes, its relation to prognosis, the relation between CD146 and EMT and the outcome to tamoxifen. Primary breast cancer tissues from 1342 patients were available for this retrospective study and immunohistochemically stained for CD146. For survival analyses, pure prognosis was studied by only including lymph-node negative patients who did not receive (neo)adjuvant systemic treatment (*n* = 551). 11% of the tumors showed CD146 expression. CD146 expression was most prevalent in triple-negative cases (64%, *p* < 0.001). In univariable analysis, CD146 expression was a prognostic factor for both metastasis-free survival (MFS) (*p* = 0.020) and overall survival (OS) (*p* = 0.037), but not in multivariable analysis (including age, tumor size, grade, estrogen receptor (ER), progesterone receptor (PR), human epidermal growth factor receptor 2 (HER2) and Ki-67). No correlation between CD146 and EMT nor difference in outcome to first-line tamoxifen was seen. In this large series, our data showed that CD146 is present in primary breast cancer and is a pure prognostic factor for MFS and OS in breast cancer patients. We did not see an association between CD146 expression and EMT nor on outcome to tamoxifen.

## 1. Introduction

CD146, also known as melanoma cell adhesion molecule (MCAM, M-CAM and MUC18), was first described in malignant melanomas as a cell adhesion molecule [1]. Since then, several different (patho)physiological roles have been described for CD146 in other types of cancer, including breast cancer. The most prominent role of CD146 in breast cancer is its involvement in the induction of epithelial-to-mesenchymal transition (EMT) [2,3,4,5]. EMT is a developmental process [6], which is frequently involved in cancer dissemination. During EMT, the morphology of the tumor cells dramatically changes, so cells gain invasive capabilities and migratory functions [7], and epithelial cell adhesion molecule (EpCAM) expression is decreased [8]. In certain instances, cancer cells also acquire stem cell-like properties after EMT [7,9]. EMT is amongst others a means for solid tumors to shed tumor cells, allowing them to intravasate into the bloodstream, where they are called circulating tumor cells (CTCs), and subsequently to extravasate to form distant metastases [10,11]. In line with its role in EMT, CD146 expression is predominantly observed in breast cancer cell lines with mesenchymal features [12,13,14]. CD146 has been associated with a poor prognosis in many types of cancer, including melanoma, prostate cancer, hepatocellular carcinoma and ovarium cancer [15,16,17,18]. Also in breast cancer, CD146 has been related to a poor prognosis [19,20], but none of the data was obtained in patients who did not receive adjuvant therapy. Furthermore, CD146 expression has been associated with high grade tumors, estrogen receptor (ER)- and progesterone receptor (PR)-negative tumors and the triple-negative subtype (ER-/PR-/ human epidermal growth factor receptor 2 (HER2)-) [3,5,19] while downmodulation of CD146 leads to a less aggressive phenotype tumor [19].

Apart from a role in tumor aggressiveness, it has been demonstrated in breast cancer cell lines that CD146 expression can confer resistance against hormonal treatment. CD146 is overexpressed in cell lines which are resistant to 4-OH-tamoxifen compared to tamoxifen-sensitive cell lines and silencing of CD146 in these resistant cell lines reversed tamoxifen resistance. In addition, in a subset of the breast cancer cell lines, CD146 expression suppressed ER expression and overexpression of CD146 in breast cancer cells induced Akt (also known as protein kinase B) activity, which is recognized as one of the mechanisms contributing to endocrine resistance [2,12,21,22]. Lastly, patients treated with adjuvant tamoxifen had significant shorter overall survival (OS), recurrence-free survival (RFS) and distant metastasis-free survival (DMFS) when CD146 expression was increased in their primary tumors [12].

Based on the above, CD146 shows promise as a useful marker for predicting disease progression and treatment response in breast cancer patients. However, regarding disease progression, previous studies included adjuvant treated patients, which clouds the issue whether or not CD146 is a prognostic or predictive marker. Thus, to gain further insight into the exact importance of CD146 in breast cancer we assessed the relation of CD146 expression, determined with immunohistochemistry (IHC), in primary breast cancer tissues with different molecular and histological subtypes. To the best of our knowledge, a pure prognostic evaluation of CD146 has not been executed. To enable this, we assessed CD146 expression in primary breast cancer tissues of patients with lymph-node negative disease who did not receive any (neo-)adjuvant systemic treatment. We also studied treatment response in a group of hormonal treatment-naive patients with recurrent breast cancer who were treated for metastatic disease with first-line tamoxifen monotherapy. In addition, EpCAM expression was determined in these tumors to assess whether gain in CD146 expression shows a loss of EpCAM expression. Lastly, we assessed the relation between CD146 expression and the expression of EMT markers at gene expression level in cell lines and primary breast cancer tissues.

## 2. Results

### 2.1. CD146 Expression and the Relationship with Patient and Tumor Characteristics

CD146 showed membranous staining >1% of the epithelial tumor cells in 113 out of the 1025 tumors (11%). In many of these tumors (*n* = 49) >50% of the tumor cells showed CD146 expression. In 17 tumors, 26–50% of the tumor cells were CD146-positive, in 23 tumors this was 11–25% and in 24 tumors 1–10% of the tumor cells. An example of positive and negative CD146 staining is depicted in supplementary Figure S1.

In Table 1, the association between the baseline characteristics of the patients and CD146 expression is shown. CD146-positive staining was more likely in tumors from patients who were younger and pre-menopausal. Also, CD146-positive tumors were associated with higher tumor grade, ER-negative, PR-negative and Ki-67-high tumors (all *p* < 0.001). Lastly, a higher T-stage at diagnosis was associated with more CD146-positive tumors (*p* = 0.016).

In the histological breast cancer subtypes (Table 2), the medullary subtype had the highest CD146 expression with 47.8% CD146-positive tumors. This subtype had significantly higher CD146 expression than tumors with an invasive lobular carcinoma (*p* < 0.001), mucinous subtype (*p* = 0.001), tubular subtype (*p* = 0.027) and invasive ductal carcinoma (*p* < 0.001). Finally, the invasive ductal carcinomas had higher CD146 expression than the invasive lobular carcinomas (*p* < 0.001).

With respect to the molecular subtypes (Table 2), the highest CD146 expression was present in the triple-negative subtype, with 63.9% CD146-positive tumors. This subtype had significantly higher CD146 expression than the other molecular subtypes (all *p* < 0.001). The HER2+ subtype has significantly higher CD146 expression than the Luminal A (*p* < 0.001), Luminal B HER2- (*p* = 0.022) and Luminal B HER2+ (*p* = 0.026) subtypes. Lastly, the Luminal B HER2- subtype had higher CD146 expression than the Luminal A subtype (*p* = 0.008).

### 2.2. CD146 and EpCAM Expression in the Diverse Breast Cancer Subtypes

Since CD146 has been associated with EMT, the expectation is that EpCAM expression is low in tumors with high CD146 expression. Of all tumors, 58% were EpCAM-high. CD146 staining was more frequently observed in EpCAM-high tumors (14.6%) compared with EpCAM-low tumors (6.0%, *p* < 0.001). In the molecular subtypes, the triple-negative group had the highest percentage of CD146-positive/EpCAM-positive staining (47.9%) and also the highest CD146-positive/EpCAM-negative staining (16.0%). For the histological subtypes, the medullary subtype had the highest percentage of CD146-positive/EpCAM-positive tumors (30.4%). For the CD146-positive/EpCAM-negative tumors, this was the papillary subtype (20.4%) (Supplementary Table S1).

### 2.3. Relationship of CD146 Expression with Expression of EMT-Related Genes

Since CD146 has the highest expression in the breast cancer cell lines with mesenchymal features, in line with its potential role in EMT, we expect that mesenchymal genes also have a higher expression in CD146-positive tumors. To study this, first the association between IHC staining of CD146 and its expression at the mRNA level (Affymetrix probe 211340_s_at) was established in a subset of 105 primary tumors. Of these patients, 25 had positive staining and showed a significant (1.7 fold) higher CD146 mRNA expression compared to patients who had CD146-negative tumors (Mann-Whitney *p* < 0.0001). Considering the relation between CD146 and mesenchymal features in breast cancer cell lines, the correlation of all genes on the array with CD146 mRNA expression was performed in 52 breast cancer cell lines. The genes with the highest correlations (R > 0.6) were reviewed in DAVID (database for annotation, visualization and integrated discovery), an online tool to discover enriched functional-related gene groups in gene lists. Indeed, EMT was a significant enriched function (multiple testing corrected *p* = 6.8 × 10^−4^). A similar analysis in a cohort of 867 primary tumors resulted in correlated genes that showed for example angiogenesis and cell adhesion as overrepresented functions, but not EMT. When comparing the genes that correlated with CD146 in both the cell lines and in the tumors, there was an overlap of 24 genes (supplementary Figure S2). These overlapping genes are amongst others, known for their function in caveolae formation and focal adhesion. Repeating the analyses for overrepresented functions in TCGA [23] and METABRIC [24] datasets also did not yield EMT as overrepresented function.

### 2.4. Relationship of CD146 Expression with Prognosis

**Figure 1:**
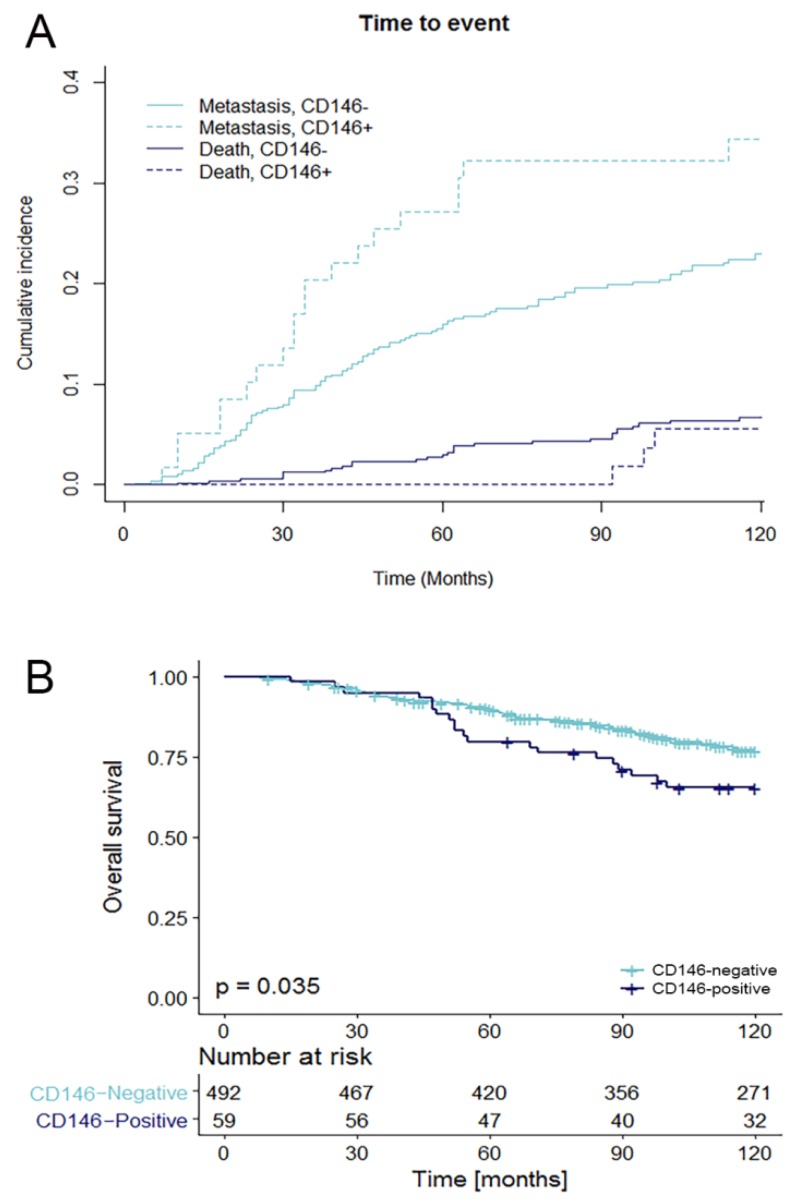
MFS and OS as function of CD146 expression. (**A**) MFS (depicted with cumulative incidence function) and (**B**) OS (depicted with Kaplan-Meier method) as function of CD146 expression. Only patients that were N0, M0 at baseline and did not receive neo-adjuvant or adjuvant therapy (*N* = 551) were included. For MFS (**A**) the patients that developed a metastasis are depicted in the light blue lines, the dark blue lines (death) are the patients that died without any evidence of disease.

For survival analysis, only patients without locoregional or distant metastases at diagnosis, N0 disease at diagnosis and who did not receive (neo-)adjuvant systemic treatment were included. Furthermore, 25 patients were lost to follow up. In total, survival analysis was performed on 551 patients. The median follow up of these patients was 98 months (range 2–334 months). For metastasis-free survival (MFS), all common prognostic factors were included (Table 3). Age > 55 year was associated with longer MFS, whereas patients with a higher tumor grade, higher T-stage and a HER2-positive or Ki-67-high tumor had shorter MFS. CD146 was associated with shorter MFS in univariable analysis (Fine & Gray model), with a hazard ratio (HR) of 1.77 (95% CI 1.09–2.87, *p* = 0.020). There was no relation between the amount (%) of CD146-positive cells and MFS. All factors in the univariable analyses are proven prognostic factors and were included in the multivariable Fine & Gray model. This model is set up to search for the best fitting model (i.e., the strongest predictive model). It showed that higher T-stage and HER2-positivity were independent predictors of shorter MFS. Age, PR-status, Ki-67-status and also CD146 (HR 1.51, 95% CI 0.79–2.87, *p* = 0.210) contribute to the quality of the model, but are not independent predictors of MFS, i.e., they make the model stronger, but are not significant predictors of MFS in multivariable analyses. The cumulative incidence function (CIF, of the Fine & Gray model) of MFS in relation to CD146 is shown in Figure 1A.

In univariable Cox regression analysis for overall survival (OS) the same prognostic factors were included (Table 4). T-stage, tumor grade, ER, PR, HER2 and Ki-67 were associated with OS. CD146 expression is associated with shorter OS in univariable analysis (HR 1.67, 95% CI 1.03–2.69, *p* = 0.037). No relation was found between the amount (%) of CD146-positive cells and OS. In the multivariable model, T-stage and HER2 remained independent prognostic factors for OS. Ki-67 was also an independent factor, but violated the proportional hazards assumption (tested with Schoenfeld residuals), and was added to the model as stratum variable. CD146 was not a significant addition to the multivariable model (HR 1.42, 95% CI 0.84–2.38, *p* = 0.191). A Kaplan-Meier curve for OS in relation to CD146 is shown in Figure 1B.

The comparison of ER-positive and ER-negative patients, showed that the ER-positive patients have a significantly shorter MFS and OS when they are CD146-positive (respectively HR 2.67, 95% CI 1.32–5.41, *p* = 0.006 and HR 2.69, 95% CI 1.30–5.55, *p* = 0.007), but in the ER-negative group there is no difference in MFS and OS between the CD146-positive and negative patients (respectively HR 1.11, 95% CI 0.51–2.45, *p* = 0.79 and HR 0.80, 95% CI 0.38–1.72, *p* = 0.57) (supplementary Figure S3). Due to small numbers, regression analysis was not performed in the other subgroups. For the number of unfavorable events in the different subgroups see supplementary Table S2.

### 2.5. CD146 Status and Outcome to Tamoxifen Treatment

The CD146 status of the primary tumor was also determined in a cohort of patients (*n* = 317) who received tamoxifen as first-line treatment in the palliative setting. All patients were ER-positive and did not receive any (neo)adjuvant endocrine treatment after diagnosis. Of these patients, only 8 patients had a CD146-positive tumor (2.5%). Although 6 out of 8 (75%) CD146-positive patients showed clinical benefit, as expected with this low number, there was no statistical difference in response on tamoxifen in the CD146-positive and -negative patients (*p* = 0.368). From the CD146-negative patients, 200 patients (65%) had clinical benefit (supplementary Table S3).

## 3. Discussion

In this study, we aimed to gain more insight into the clinical significance of CD146 expression in breast cancer and to determine if CD146 is a pure prognostic marker. We showed that 11% of the primary breast cancer tissues express CD146. This is in line with a previously published paper, in which 7% of all tumors (*n* = 635) had CD146 expression [19], though in another study a percentage of 35% CD146-positive tumors was described (*n* = 505), but this study contained 29% triple negative tumors, while in our study only 11% was triple-negative [3].

In literature, CD146 expression was predominantly seen in breast cancer cell lines with mesenchymal features [12,13,14]. In our study, we could confirm this and additionally found a relation between CD146 and EMT in the breast cancer cell lines. It is also known that CD146 expression varies across the different breast cancer subtypes, especially the triple-negative breast cancer subtype has been associated with CD146 expression [3,5]. Our data confirms that CD146 expression is more prevalent in triple-negative breast cancers than in other breast cancer subtypes (63.9% vs. 4.1%). The triple-negative subtype can be divided into six different subtypes, of which one is the mesenchymal subtype [25]. However, in the primary breast cancer tumors of this dataset and in the publicly available TCGA and METABRIC data, there was no correlation between genes that are associated with EMT and CD146 expression. Another observed phenomenon of tumors that undergo EMT is loss of EpCAM [8]. Therefore, we expected tumors with CD146 expression to have loss of EpCAM. Our data did not show an inverse relationship between CD146 and EpCAM expression in any of the investigated subtypes. CD146 expression was even more frequently observed in EpCAM-high compared to EpCAM-low tumors. Thus, while in cell lines it is clear there is an association between CD146 and EMT, it is not clear-cut that CD146 expression is indicative for EMT in primary breast cancers. In previously published papers, CD146 expression has also been linked to EMT in cell lines and mouse models [2,3], but our data cannot confirm this relation between CD146 expression and EMT in primary breast cancers. Therefore, while others have shown in preclinical experiments that CD146 expression is necessary for the induction of EMT, our data in primary breast tumors suggests that CD146 expression alone is insufficient to drive a breast cancer tumor cell into EMT.

With respect to the histological subtypes, the medullary subtype had the highest CD146 expression. Of the 23 medullary tumors included in this study, 11 of these tumors were of the triple-negative molecular subtype. So more than half of the medullary tumors are of another molecular subtypes (mainly luminal B HER2-subtype (*n* = 7)).

In other tumor types, CD146 expression is associated with tumors with a higher tumor grade and an increased metastatic potential [18,26,27]. The data shown here confirms that this is also the case in breast cancer. In addition, CD146 expression was more prevalent in tumors from younger patients. Regarding prognosis, this data shows that CD146 is a pure prognostic factor in the first 10 years of follow-up, a result not confounded by including patients who were treated with adjuvant systemic therapy, as others have done previously. In the study of Zabouo et al. [19], 51% of the patients received adjuvant chemotherapy and 52% adjuvant endocrine therapy, which obscures the assessment of the true prognostic value of a marker. It is not clear if the patients in the study of Zeng et al. [3] received any type of systemic (neo)adjuvant treatment. While Zeng et al. also showed worse MFS and OS for CD146-positive patients with a follow-up period of 100 months, Zabouo et al. showed that CD146-positive patients had significantly lower OS in the first 5 years, but not in the period thereafter [3,19]. The data presented here (Figure 1) shows that the effect of CD146 expression is more distinct as time progresses for OS. OS does not differ in the first 45 months with respect to CD146, but between 45 and 120 months the CD146-positive patients have a worse prognosis (HR 2.16, 95% CI 1.25–3.74, *p* = 0.005, see Supplementary Figure S4). When correcting for other prognostic factors of breast cancer in multivariable analysis, which has not been done before, CD146 does add to the quality of the model for MFS, but it is not a significant independent prognostic factor for MFS nor OS.

We divided the group of patients in an ER-positive and ER-negative subgroup to explore what the difference in prognostic impact of CD146 is between these two subgroups. It is striking that in the ER-negative subgroup, there is no difference in MFS and OS between the CD146-positive and CD146-negative patients, while in the ER-positive subgroup both MFS and OS are shorter in the CD146-positive group, although the numbers of CD146-positive patients is somewhat low. In supplementary Figure S3 it was found that 80% of the ER-positive, CD146-negative patients were alive after 10 years, while the CD146-positive patients, with probably more aggressive tumors, only 53% were still alive. In the ER-negative patients this was around 70% for both the CD146-positive and -negative subgroup. So, although CD146 expression is rare in ER-positive patients, CD146 has the potential to be of value as a potential prognostic marker in this subgroup. It should be recommended to validate this in a larger independent series, preferably prospectively, since this could potentially have consequences for ER-positive/CD146-positive patients, for example in more extensive monitoring or prolonging of the duration of adjuvant therapy.

As shown previously, CD146 expression is associated with tamoxifen resistance in breast cancer cell lines [12]. To examine whether this also holds true in patients, we investigated this in a set of primary breast cancer tissues of patients who did not receive (neo)adjuvant endocrine treatment and were treated in the first-line palliative treatment with tamoxifen. In total, only 2.5% (8/317) of these patients were CD146-positive. Though no difference in outcome to tamoxifen was seen in this set of patients, the number of CD146-positive patients is too small to draw firm conclusions.

CD146 expression is present in 11% of all primary breast cancer tissues and is predominantly present in the medullary and triple-negative subtypes. We found no strong evidence of CD146 expression and EMT in primary breast tumors, suggesting that CD146 is maybe necessary, but not sufficient for EMT in breast cancer. CD146 expression was associated with more aggressive tumors and patients who are CD146-positive had a shorter MFS and OS, without the confounding effect of adjuvant treatment.

## 4. Materials and Methods

### 4.1. Patient and Tissue Samples 

**Figure 2:**
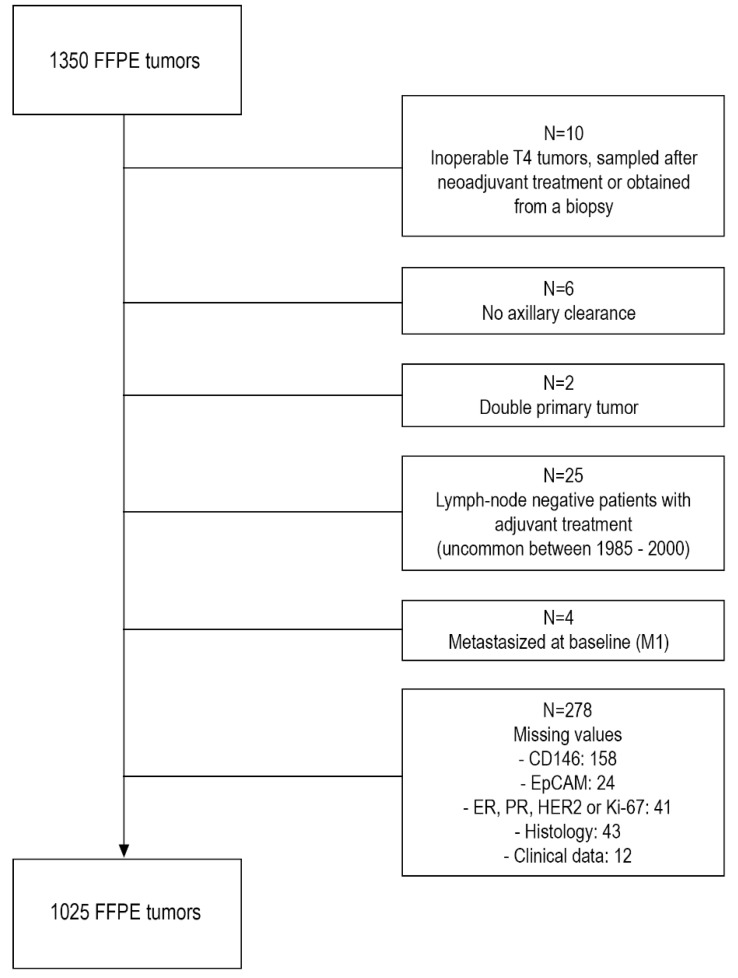
Flowchart of formalin-fixed paraffin-embedded (FFPE) tissues included in the study (*n* = 1350).

Formalin-fixed paraffin-embedded (FFPE) tissue of the primary tumor was collected from all patients with breast cancer who entered the Erasmus University Medical Center (Rotterdam, The Netherlands) for local treatment of their primary disease during the period of 1985 to 2000. This study was approved by the Erasmus MC medical ethics committee (MEC 02-953, approved 11th of April 2002). In total, from 1350 patients FFPE tissues and complete clinical follow-up information was collected for the primary objective. Due to missing values and several other reasons that are listed in Figure 2, 325 tissues were excluded, leaving a total of 1025 FFPE tissues for analysis. All tissues had a known histological subtype and molecular surrogate subtype and could therefore be used for subtype analysis. 551 tissues were from patients who were lymph-node negative and did not receive (neo-)adjuvant treatment and hence could be used to assess the pure prognostic value of CD146. To assess the role of CD146 in response to tamoxifen treatment, an additional set of 462 FFPE tissues of the primary tumor of breast cancer patients who received tamoxifen as first-line palliative treatment was collected. Of these, CD146 status was determined of 317 ER-positive tumors from patients who did not receive (neo)adjuvant endocrine treatment.

### 4.2. Tissue Microarray and Immunohistochemistry

Pathologists (MdB, CvD) from the Erasmus Medical Center assessed the histology of all eligible tumors and also scored tumor grade according to the modified method of Scarff, Bloom & Richardson [28] prior to preparing the tissue microarray (TMA). Thereafter, representative areas of the tumor for inclusion in the TMA were marked. From these areas, three cores were taken with a 0.6mm needle and added to the TMA. These TMAs were stained with standard protocols for ER, PgR, HER2, Ki-67, EpCAM and CD146 and scored manually for staining intensity (0 = negative, 1 = weak, 2 = moderate, 3 = strong) and quantity (percent of stained breast tumor cells). See Supplementary Table S4 for more information about IHC staining methods, cut-offs and used subtypes [29,30].

### 4.3. Epithelial-to-Mesenchymal Transition 

To study the role of CD146 in EMT, gene expression data was used of 52 breast cancer cell lines (available as entry GSE41313at the Gene Expression Omnibus (GEO) http://www.ncbi.nlm.nih.gov/ geo/), 105 tissues from our dataset and 867 primary breast cancer tumors (in-house data plus publicly available data. GEO entries GSE2034, GSE5327, GSE12276, GSE27830 and GSE47389 (in-house) and GSE2990, GSE7390 and GSE11121). The data were normalized using fRMA [31] and were corrected for batch effects using ComBat [32] before analyses. First, correlation of all available probes (plus2-PM chip, Affymetrix, Santa Clara, CA, USA) to the CD146 probe 211340_s_at was assessed in the 52 cell lines. An arbitrarily chosen cutoff of R > 0.6 was used to ensure both a decent correlation with CD146 and a sufficient number of genes (*n* = 342) were then available for overrepresentation analysis using DAVID [33,34]. The same analysis was performed in the primary tumor cohort. However, since correlations in the tumor cohort ranged between −0.35 and +0.6, another cutoff was established. The correlation coefficients were normally distributed and the average and standard deviation (SD) were used to calculate a cutoff for the extreme end of the distribution (*p* < 0.001). This gave an R of 0.35 and yielded 200 genes for overrepresentation analysis. This method was also used for TCGA [23] and METABRIC [24] data (downloaded from cBioPortal—http://www.cbioportal.org/), yielding a cutoff of R > 0.502 and 37 genes for analysis (TCGA) and R > 0.276 and 47 genes (METABRIC). So although the absolute correlation is not that high in the primary tumor data, the selected genes are the highest correlating genes with CD146.

### 4.4. Statistical Analysis 

The relation between patient characteristics and the immunohistochemical profile of the tumor were analyzed with the Pearson chi-square test. For subtype analysis, the Fisher’s exact test is used in case of small subtype groups. For all patients, time from diagnosis to the first distant metastasis (MFS) and time from diagnosis until patient’s death (OS) was determined. Patients who did not experience an event were censored at the last date of contact (with a maximum of 10 years, since patients usually return to the general practitioner for follow-up after 10 years). For MFS, all distant metastases were counted as an event, but local-regional relapses were not. Patients diagnosed with secondary contralateral breast cancer were censored for MFS at the date of diagnosis of the secondary breast cancer. A total of 97 patients died without evidence of disease and were censored at last follow-up in the analysis of MFS. During follow up, 316 patients died with evidence of disease. To assess the prognostic role of CD146, only data from patients who were lymph-node negative and did not receive (neo)adjuvant treatment were used (*n* = 551). For the patients who were treated with tamoxifen, clinical benefit (CB) is defined as patients that show complete response (CR), partial response (PR) or stable disease (SD) for more than 6 months on tamoxifen treatment. Survival analyses for MFS were performed using the competing risks model of Fine & Gray to account for the competing risk of death of patients who did not have evidence of disease at the moment of death. MFS is visualized by means of the cumulative incidence function (CIF). For OS the Cox proportional hazards model was used and the Kaplan-Meier method for visualization. All factors from the univariable analyses were added to the multivariable analysis where a stepwise backward selection procedure was used to study which factors are independently related to the outcomes. In the Cox models this was performed with a threshold for significance of *p* < 0.05. For the Fine & Gray model this procedure was based on the Akaike information criterion (AIC). This method is not *p*-value driven, but compares the relative quality of the models with different combinations of prognostic factors to each other and selects the best fitting model. The results are presented with a hazard ratio (HR) and its 95% confidence interval (95% CI). All computations were performed using R and all reported *p*-values are two-sided.

## Figures and Tables

**Table 1:** Baseline characteristics and the relation to CD146 expression (*n* = 1025).

Characteristics	*N*	CD146-Negative	CD146-Positive	*p*-Value
Age				*p* < 0.001 *
≤40	127	96	31	
41–55	431	383	48	
≥55	467	433	34	
T-Stage				*p* = 0.016
T1	602	547	55	
T2–T4	408	351	57	
N-Stage				*p* = 0.577 *
N0	576	511	65	
N1	347	308	39	
N2	102	93	9	
Menopausal status				*p* < 0.001
Pre-menopausal	477	405	72	
Post-menopausal	548	507	41	
Tumor grade				*p* < 0.001 *
Grade I	211	205	6	
Grade II	473	451	22	
Grade III	341	256	85	
Ki-67 status				*p* < 0.001
Low (<10%)	621	595	26	
High (≥10%)	404	317	87	
ER status				*p* < 0.001
Positive	861	831	30	
Negative	164	81	83	
PR status				*p* < 0.001
Positive	657	639	18	
Negative	368	273	95	
HER2 status				*p* = 0.200
Positive	119	110	9	
Negative	906	802	104	

Chi-square test performed for tumor grade, N-stage and Age (*): test for trend performed. In the T-stage analysis 15 tissues were removed due to unknown T-stage status. Of these 14 were CD146-negative and one was CD146-positive.

**Table 2:** CD146 expression in histological and molecular breast cancer subtypes.

CD146 Expression			
Tumor types	Negative *N* (%)	Positive *N* (%)	*p*-Value
All tumors (*n* = 1025)	912 (89.0)	113 (11.0)	
Histological subtype			*p* < 0.001
Invasive ductal carcinoma	751 (88.5)	98 (11.5)	
Invasive lobular carcinoma	117 (99.2)	1 (0.8)	
Medullary	12 (52.2)	11 (47.8)	
Mucinous	18 (100)	0 (0.0)	
Tubular	11 (91.7)	1 (8.3)	
Papillary	3 (60.0)	2 (40.0)	
Molecular subtype			*p* < 0.001
Luminal A	437 (98.0)	9 (2.0)	
Luminal B HER2-negative	322 (94.4)	19 (5.6)	
Luminal B HER2-positive	72 (97.3)	2 (2.7)	
HER2-positive	38 (84.4)	7 (15.6)	
Triple negative	43 (36.1)	76 (63.9)	

Number and percentage of CD146-positive and -negative tumors. Also divided by histological and molecular subtypes. The reported *p*-values are the comparison within the molecular subtypes and within the histological subtypes.

**Table 3:** Fine & Gray regression analysis for metastasis-free survival (MFS).

Characteristics	Univariable Analysis			Multivariable Analysis		
	HR	95% CI	*p*-Value	HR	95% CI	*p*-Value
Age						
40–55 vs. <40	0.69	0.41–1.17	0.170			
>55 vs. <40	0.48	0.28–0.81	0.006	0.63	0.35–1.15	0.130
T-stage						
T2–T4 vs. T1	1.94	1.36–2.76	<0.001	1.77	1.22–2.59	0.003
Tumor grade						
II vs. I	2.44	1.38–4.30	0.002			
III vs. I	2.94	1.64–5.27	<0.001			
ER						
Pos vs. neg	0.74	0.47–1.18	0.210			
PR						
Pos vs. neg	0.85	0.59–1.23	0.390	1.29	0.83–1.99	0.260
HER2						
Pos vs. neg	2.92	1.92–4.46	<0.001	2.64	1.65–4.22	<0.001
Ki-67						
Pos vs. neg	1.82	1.28–2.59	<0.001	1.37	0.92–2.05	0.120
				Additions to the base model		
CD146						
Pos vs. neg	1.77	1.09–2.87	0.020	1.51	0.79–2.87	0.210

Univariable and multivariable regression analysis for MFS during 120 months of follow up. Only patients that were N0, M0 at baseline and did not receive neo-adjuvant or adjuvant therapy (*N* = 551) were included.

**Table 4:** Cox regression analysis for overall survival (OS).

Characteristics	Univariable Analysis			Multivariable Analysis		
	HR	95% CI	*p*-Value	HR	95% CI	*p*-Value
Age						
40–55 vs. <40	0.68	0.39–1.18	0.170			
>55 vs. <40	0.76	0.44–1.30	0.311			
T-stage						
T2–T4 vs. T1	1.98	1.39–2.83	<0.001	1.86	1.30–2.66	<0.001
Tumor grade						
II vs. I	2.06	1.19–3.59	0.010			
III vs. I	2.51	1.43–4.41	0.001			
ER						
Pos vs. neg	0.63	0.41–0.97	0.035			
PR						
Pos vs. neg	0.63	0.44–0.90	0.011			
HER2						
Pos vs. neg	2.55	1.68–3.87	<0.001	2.15	1.39–3.31	<0.001
Ki-67						
Pos vs. neg	1.71	1.20–2.43	0.003			
				Additions to the base model		
CD146						
Pos vs. neg	1.67	1.03–2.69	0.037	1.42	0.84–2.38	0.191

Univariable and multivariable regression analysis for OS during 120 months of follow up. Only patients that were N0, M0 at baseline and did not receive neo-adjuvant or adjuvant therapy (*N* = 551) were included. The multivariable regression analysis has been stratified for Ki-67, both the base model as the base model with addition of CD146.

## Supplementary Materials

The following are available online at http://www.mdpi.com/2072-6694/10/5/134/s1, Supplementary Figure S1: Immunohistochemical staining for CD146; Supplementary Figure S2: Overlapping genes between cell lines and primary tumors; Supplementary Figure S3: MFS and OS as function of CD146 expression in the ER-positive and -negative subgroup; Supplementary Figure S4: OS as function of CD146 expression divided in two time periods; Supplementary Table S1: Relationship between EpCAM and CD146 expression; Supplementary Table S2: Number of unfavorable events in the histological and molecular breast cancer subtypes; Supplementary Table S3: CD146 status in relation to the type of response on tamoxifen in the first line; Supplementary Table S4: Methods of the TMA immunohistochemistry and breast cancer subtypes.
