# Supplementary material for: The Prevalence of CD146 Expression in Breast Cancer Subtypes and Its Relation to Outcome
Source: Cancers (Basel). 2018 May 5;10(5):134. doi: 10.3390/cancers10050134 (PMC5977107; doi:10.3390/cancers10050134)
Supplement: Supplementary file 1 [file cancers-10-00134-s001.zip › Titles and legends to supplementary figures - adjusted after proofs.docx]

**Supplementary figure 1: Immunohistochemical staining for CD146**

*Immunohistochemical staining of CD146 in a A) CD146-positive and B) CD146-negative primary breast cancer tumor (only CD146 staining in vessels)*

**Supplementary figure 2: Overlapping genes between cell lines and primary tumors**

*From the highest correlating genes in the cell lines and the primary tissues, these are the 24 overlapping genes.*

**Supplementary figure 3: MFS and OS as function of CD146 expression in the ER-positive and negative subgroup**

*MFS and OS as function of CD146 expression. A) MFS in ER-positive subgroup and B) ER-negative subgroup (depicted with the cumulative incidence function). C) OS in ER-positive subgroup and D) ER-negative subgroup (depicted with Kaplan-Meier). For A) and B) the light blue lines (metastasis) are the patients that developed a metastasis, the dark blue lines (death) are the patients that died without any evidence of disease.*

**Supplementary figure 4: OS as function of CD146 expression divided in two time periods**

*OS (depicted with Kaplan-Meier method) as function of CD146 expression in patients that were N0, M0 at baseline and did not receive neo-adjuvant or adjuvant therapy (N = 551). A) shows the OS of these patients in the first 45 months (months 0-45) and B) the OS from month 46 till 120 (months 46-120).*
