# Supplementary figures and images for: The Prevalence of CD146 Expression in Breast Cancer Subtypes and Its Relation to Outcome
Source: Cancers (Basel). 2018 May 5;10(5):134. doi: 10.3390/cancers10050134 (PMC5977107; doi:10.3390/cancers10050134)

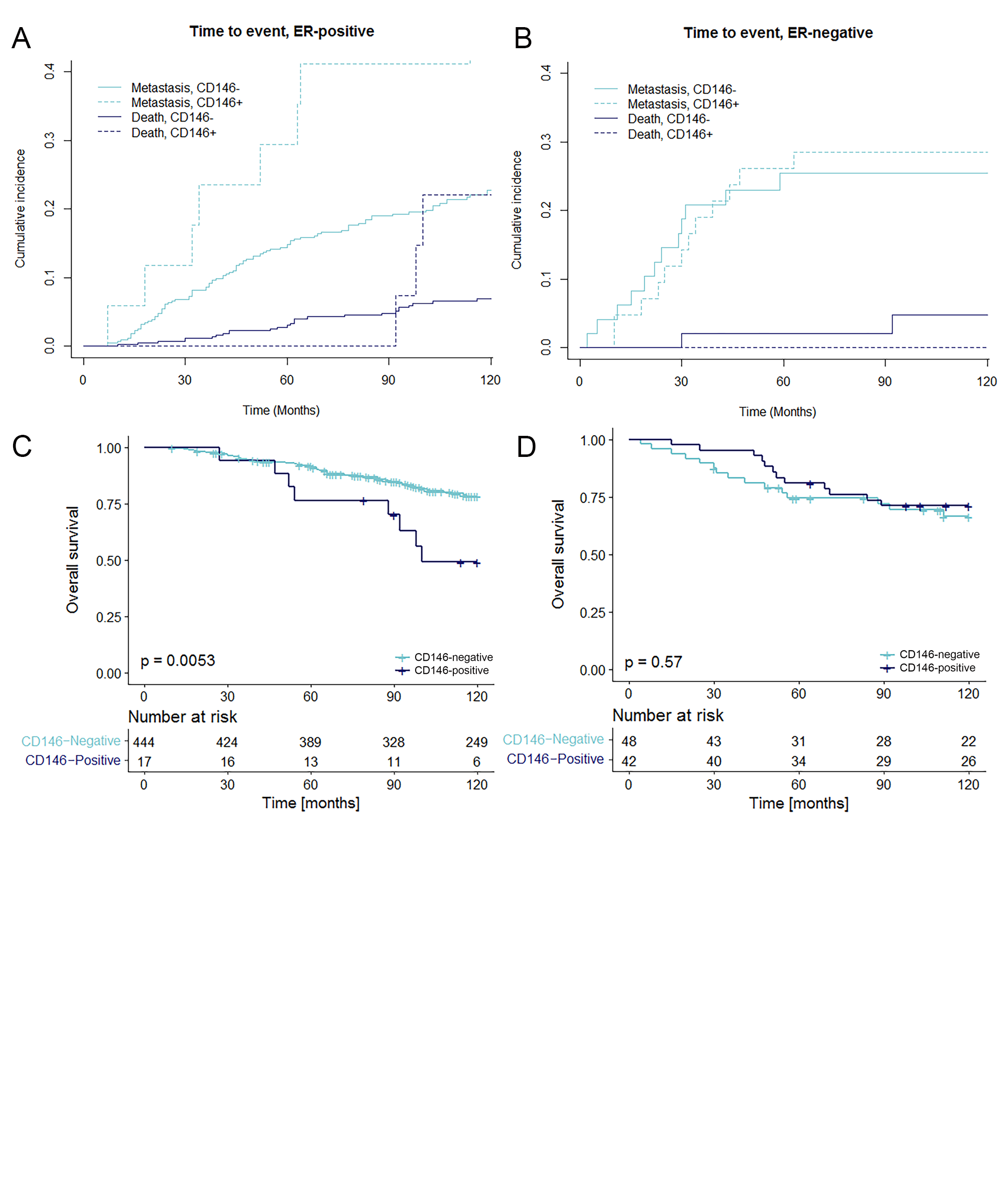

Supplement: Supplementary file 1 [file cancers-10-00134-s001.zip › Sup. figure 3 - adjusted after proofs.tif]

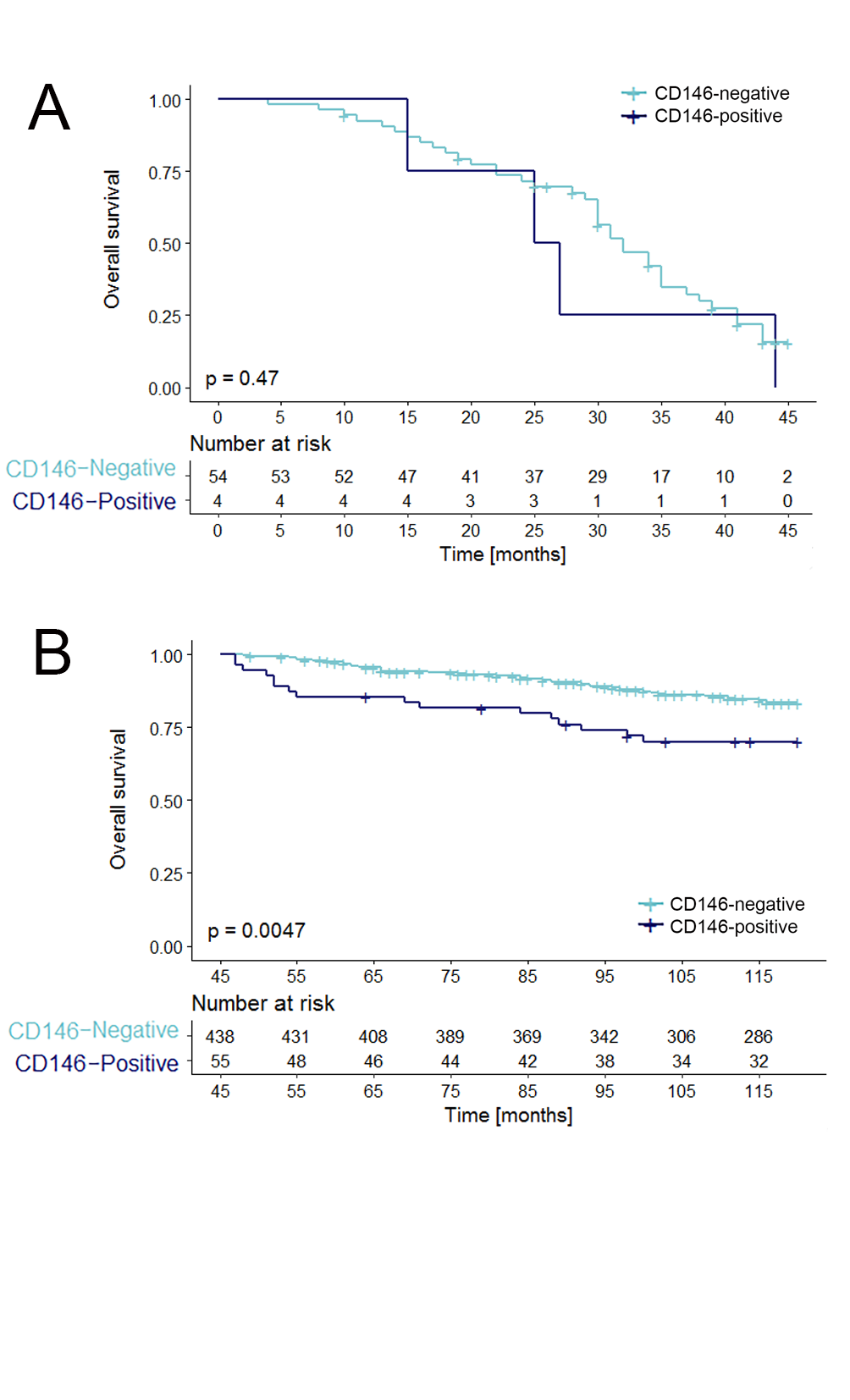

Supplement: Supplementary file 1 [file cancers-10-00134-s001.zip › Sup. figure 4 - adjusted after proofs.tif]
